# A century-long eddy-resolving simulation of global oceanic large- and mesoscale state

**DOI:** 10.1038/s41597-022-01766-9

**Published:** 2022-11-11

**Authors:** Mengrong Ding, Hailong Liu, Pengfei Lin, Yao Meng, Weipeng Zheng, Bo An, Yihua Luan, Yongqiang Yu, Zipeng Yu, Yiwen Li, Jinfeng Ma, Jian Chen, Kangjun Chen

**Affiliations:** 1 State Key Laboratory of Numerical Modeling for Atmospheric Sciences and Geophysical Fluid Dynamics, Institute of Atmospheric Physics, Chinese Academy of Sciences, Beijing, 100029 China; 2 Marine Science and Technology College, Zhejiang Ocean University, Zhoushan, 316022 China; 3 College of Earth and Planetary Sciences, University of Chinese Academy of Sciences, Beijing, 100049 China; 4 Center for Ocean Mega-Science, Chinese Academy of Sciences, Qingdao, 266071 China; 5 Centre for Geography and Environmental Science, University of Exeter, Penryn Campus, Cornwall, TR10 9FE UK; 6 Earth System Numerical Simulation Science Center, Institute of Atmospheric Physics, Chinese Academy of Sciences, Beijing, 100029 China; 7 Beijing Institute of Applied Meteorology, Beijing, 100089 China; 8 Present Address: Earth System Numerical Simulation Science Center, Institute of Atmospheric Physics, Chinese Academy of Sciences, Beijing, 100029 China

**Keywords:** Physical oceanography, Climate and Earth system modelling

## Abstract

Investigating oceanic variations at multiple spatial and temporal scales is vital for an in-depth understanding of the ocean response to global climate change. However, the available observational datasets contain uncertainties and deficiencies that leave them insufficient for investigating global ocean variability with long temporal scales and/or meso spatial scales. Here, we present a daily and century-long (1901–2010) global oceanic simulation dataset with high resolution (1/10° horizontal resolution and 55 vertical layers) forced by 6-hour atmospheric data from ERA-20C. Preliminary evaluations demonstrate that this simulation can realistically reproduce the large-scale global ocean circulation and capture the essential features of global surface mesoscale eddies. This long-running high-resolution simulation dataset provides temporally highly resolved oceanic and flux variables. Together with its good performance in simulating the global oceanic state, this eddy-resolving simulation has the potential to help toward a better understanding of ocean variability at multiple spatial and temporal scales.

## Abstract (summary)

**Table:** 

Measurement(s)	ocean
Technology Type(s)	Model
Factor Type(s)	Sea Surface Height Above Geoid • Sea Water Surface Downward X Stress • Sea Water Surface Downward Y Stress • Downwelling Shortwave Radiation in Sea Water • Surface Upwelling Longwave Radiation • Ocean Mixed Layer Thickness Defined by Sigma T • Water Flux into Sea Water • Sea-Ice Area Percentage • Surface Upward Sensible Heat Flux • Sea Water Potential Temperature • Sea Water Salinity • Sea Water X velocity • Sea Water Y velocity • Sea Water Vertical Velocity
Sample Characteristic - Environment	ocean
Sample Characteristic - Location	global ocean

## Background & Summary

The ocean plays a key role in mitigating climate change by serving as a heat and carbon reservoir^1–3^. Over the past 50 years, more than 90% of the additional heat trapped in the earth system by the increasing concentrations of greenhouse gas have been stored in the ocean^4–7^. The continuously warming ocean results in sea-level rise^8–10^, ocean acidification^11–13^, and strengthening of tropical storms^14–16^. In turn, all these effects have the potential to substantially change the daily life of human beings by altering weather and climate patterns, particularly in coastal regions^10,17,18^.

In response to climate change, ocean dynamics on large and mesoscales, both globally and regionally, have been substantially altered^19–28^. However, due to the insufficient spatial resolution and limited time length of the observational datasets, the global, multi-decadal variabilities of large-scale circulation and mesoscale eddies have not yet been sufficiently understood. In particular, most mesoscale analyses are more focused on the regional variabilities^23–26^ and greatly depend on satellite datasets^25–27^. Variabilities of global mesoscale eddies beyond the satellite period remain poorly recognized.

Recent studies have indicated that the effect of mesoscale eddies on the spatial and temporal variabilities of the large-scale circulation should not be neglected^29–33^. Mesoscale eddies play a potential role in determining the strength of the gyre circulations and their low-frequency variabilities^29–32^. Over the Southern Ocean, eddy-induced transport can partially cancel out the wind-driven meridional overturning circulation due to the increased surface-wind work^33^. The compensation effects also lead to the insensitivity of the upper-layer circulation to the surface-wind changes in this region. More examples can be found in recent review papers^34,35^. Therefore, understanding global ocean eddy dynamics and its role in influencing multi-decadal variabilities of large-scale circulation is of scientific interest.

The numerical simulations of Ocean General Circulation Models (OGCMs)^36–40^ and (or) reanalysis data^41,42^ are essential data sources for research on climate change, apart from *in-situ* observations and satellite datasets. These products—for example, the model outputs from the Coupled Model Intercomparison Project (CMIP)^43,44^ and MIPs for the ocean-only models (OMIPs)^45–47^—can extend the satellite epoch and be applied in various research efforts on past climate change^48,49^ and future climate prediction^50,51^. Besides, unlike the satellite observations that are limited to the sea surface, OGCMs have the advantage of simulating all the three-dimensional properties beneath the sea surface^52,53^.

With the tremendous increase in computing resources, the eddy-resolving resolution (approximately 10 km) has started to be employed for ocean-only^46,47^ and coupled models^34,54^. Consequently, the capability of OGCMs to reproduce the large-scale^46^ and mesoscale^46,47^ ocean dynamics, energy cascade between different scales^55^, air–sea interaction^56^, and ocean heat uptake^57^ has dramatically improved. Recently, simulations using higher-resolution OGCMs are beginning to emerge^58,59^. The increasing horizontal resolution effectively refines the simulation performances of OGCMs^47,54^ and makes them available to predict ocean dynamics for both large-scale circulation and mesoscale eddies^34,60^. The application of long-term eddy-resolving simulations can provide more possibilities to further study the ocean variability at multiple spatial and temporal scales.

Here, we present a 110-year (1901–2010) eddy-resolving simulation following the OMIP protocol. It is forced by the 6-hour atmospheric data from the European Centre for Medium‐Range Weather Forecasts (ECMWF) twentieth century reanalysis (ERA-20C)^41^. Preliminary evaluation shows that this dataset performs excellently in simulating the mean large-scale circulation. The spatial pattern and temporal evolution of large-scale climate modes is reproduced as well. The eddy-resolving resolution makes it possible for this simulation to capture the statistical characteristics of mesoscale eddies in the tropics and subtropics. We believe the application of this simulation dataset will be of great importance in understanding the ocean dynamics on both large and mesoscales under global warming. This century-long eddy-resolving simulation dataset can also be used as a high-resolution supplementary data source for data-driven studies^61,62^.

## Methods

### Model and its configuration

The model is the State Key Laboratory of Numerical Modeling for Atmospheric Sciences and Geophysical Fluid Dynamics, Institute of Atmospheric Physics (LASG/IAP) Climate System Ocean Model version 3 (LICOM3). LICOM3 was substantially improved compared to its predecessor, LICOM2.0^63^, including an upgrading of the coupling interface from National Center for Atmospheric Research (NCAR) flux coupler version 6 to 7^64^, the introduction of arbitrary orthogonal curvilinear coordinates^65^, and an updating of the physical package, such as the vertical mixing due to the internal tides breaking at the bottom^66^ and a spatially and temporally varying thickness diffusivity^67^. Both the low (1°)^68^ and high (1/10°)^40^ resolutions of LICOM3 have been involved in OMIP1 and OMIP2. In addition, the 1/10° version has been applied to perform short-term ocean forecasts for operational purposes^60^.

In this study, we chose the version of LICOM3 with the eddy-resolving resolution (1/10°)^40^ for the century-long experiment. The global mesh has 3600 × 2302 horizontal grid cells, with a zonal cell size of about 11 km at the equator to 8 km at midlatitudes. Vertically, there are 55 levels, with 5 m in the uppermost level using the η coordinate^40^. The experiment follows the OMIP protocol^45^, except that the forcing dataset is derived from the 6-hour data from ERA-20C^41^. It is important to note that the wind stress is calculated based on the relative winds. In addition, LICOM3 is coupled with the thermodynamic sea-ice model of the Community Ice Code version 4^69^ through NCAR flux coupler 7.

### Experimental design

The experiment follows the OMIP-2 protocol^45^ but is forced by ERA-20C dataset and initialized from a 60-year spin-up experiment of the high-resolution version of LICOM3. The spin-up experiment starts from a resting ocean with the temperature and salinity from the Mercator Ocean analysis dataset^42^ on 1 January 2014. The 6-hourly ERA-20C dataset in 1901 is employed to force the model repeatedly for 60 years. The model outputs on 31 December of the 60th model year are used as the restart file to further initialize the experiment. The model has a quasi-equilibrium circulation, particularly in the upper wind-driven regime (figure not shown). After that, the eddy-resolving LICOM3 simulation is integrated for another 110 years from 1901 to 2010, forced by the 6-hourly ERA-20C data, except for the runoff data, which are from the climatological CORE II dataset^70^. The atmospheric state variables include 10 m air temperature, 10 m relative humidity, 10 m vector winds, sea level pressure, and precipitation, as well as two radiative fluxes: downward shortwave radiation and downward longwave radiation. The daily averaged variables stored and analyzed are over the whole period of integration (1901–2010), including the surface heat, freshwater and momentum fluxes, the sea surface height, and three-dimensional temperature, salinity, and ocean currents on a tripolar Arakawa B grid.

## Data Records

### Format of data

This dataset includes the model output and the derived global mesoscale eddy dataset. The former contains the model output of nine two-dimensional and five three-dimensional ocean state and surface flux variables at 0.1° resolution. All stored variables are listed in Table 1. The netCDF4 CF-compliant files we uploaded were created by Climate Model Output Rewriter version 3 (CMOR3) and the metadata they contain fulfill the requirements of the MIPs.

**Table 1:** Descriptions of data variables.

	Variable name	Long name	Unit	Spatial/Temporal resolution
2D	zos	Sea Surface Height Above Geoid	meter	0.1° × 0.1°/daily
	tauuo	Sea Water Surface Downward X Stress	N/m^2^	
	tauvo	Sea Water Surface Downward Y Stress	N/m^2^	
	rsdo	Downwelling Shortwave Radiation in Sea Water	W/m^2^	
	rlus	Surface Upwelling Longwave Radiation	W/m^2^	
	mlotst	Ocean Mixed Layer Thickness Defined by Sigma T	meter	
	wfo	Water Flux into Sea Water	kg/m^2^/s	
	siconc	Sea-Ice Area Percentage	%	
	hfsso	Surface Upward Sensible Heat Flux	W/m^2^	
3D	thetao	Sea Water Potential Temperature	^°^C	
	so	Sea Water Salinity	0.001	
	uo	Sea Water X velocity	m/s	
	vo	Sea Water Y velocity	m/s	
	wo	Sea Water Vertical Velocity	m/s	

Besides the model output, a global mesoscale eddy dataset based on these simulation data is provided within the dataset. The global mesoscale eddies with lifetimes longer than 28 days are identified based on the sea surface height contour automated algorithm^71–74^ and tracked by the modified genealogical evolution model^73,74^. The eddy trajectory data provide the longitude and latitude of the eddy’s centroid, the kinematic metrics when the eddies are identified, including the eddy amplitude and radius, and the eddy lifetime. The eddy amplitude is defined as the absolute difference of in the sea surface height anomalies anomaly between the outermost closed contour and the maximum/minimum at the eddy center, and the eddy radius is defined as the radius of a circle whose area is equal to the area enclosed by the eddy perimeter.

### Access to the data

The data files are provided in netCDF-4 classic format and archived in the Science Data Bank (10.11922/sciencedb.j00076.00095^75^). In this repository, we offer the daily model outputs from 1993 to 2010 at 0.1° resolution, the daily sea surface height and the monthly model outputs from 1901 to 2010 interpolated to 0.25°resolution, and the global eddy trajectories along with the data used to generate the figures in this paper. To ease the load of the data repository, the daily three-dimensional variables in the upper 1000 m only (42 levels) are provided. The current total data volume of the entire dataset is approximately 25 TB.

## Technical Validation

To assess the dataset in reproducing the variabilities at multiple spatial and temporal scales, we present three aspects of the simulated results in this section. First, the global statistics obtained from the century-long simulation are evaluated against available observations and/or reanalysis datasets. Second, the simulated and observed results are compared to evaluate the reproducibility of the stationary and variable ocean dynamics. And third, the global oceanic coherent eddy features in this simulation highlight the performance in reproducing oceanic mesoscale phenomena.

### Temporal evolution of global diagnostics

**Fig. 1:**
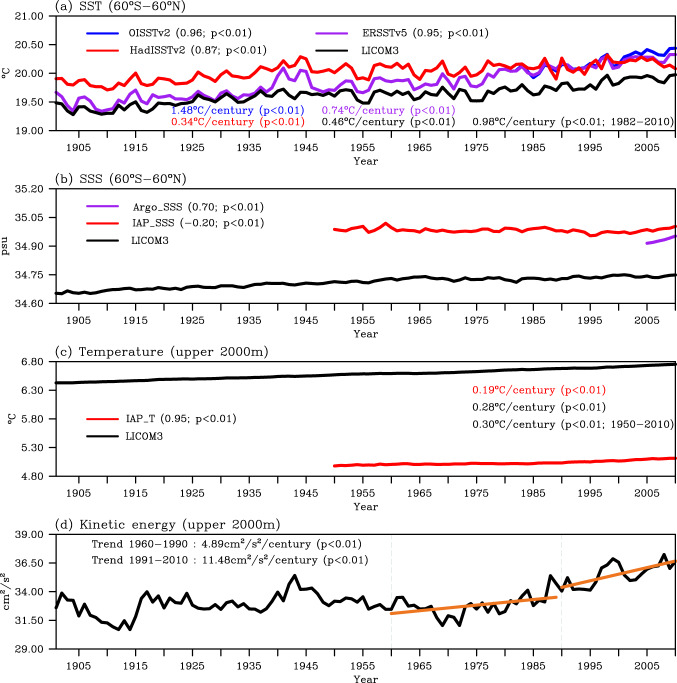
Globally averaged yearly mean (**a**) sea surface temperature (SST, °C), (**b**) sea surface salinity (SSS, psu), (**c**) integrated ocean temperature in the upper 2000 m (°C), and (**d**) integrated kinetic energy in the upper 2000 m (cm^2^/s^2^) for the LICOM3 simulation (black). The observations in (**a–c**) are denoted by color lines [(**a**) blue for OISSTv2, purple for ERSSTv5, and red for HadISSTv2; (**b**) purple for Argo_SSS, red for IAP_SSS; (**c**) red for IAP_T]. The temporal correlation coefficients between the LICOM3 simulation and observations and the corresponding *p*-values (99% confidence level is exceeded when *p* < 0.01) are labeled in the legend in (**a–c**). The linear trends of SST and integrated ocean temperature in the upper 2000 m are also marked in (**a**) and (**c**), respectively. The orange lines in (**d**) are the linear fitting lines of oceanic kinetic energy during 1960–1990 and 1991–2010, and the linear trends are marked in the upper left.

The temporal evolution of the upper-ocean circulation measured by four quasi-global averaged metrics—namely, the averaged sea surface temperature (SST) and sea surface salinity (SSS) within 60°S–60°N, and the averaged ocean temperature and kinetic energy in the upper 2000 m of ocean within 60°S–60°N—are evaluated against the observational and/or reanalysis datasets (Fig. 1 and Table 2).

**Table 2:** Observational or reanalysis datasets used in this study.

Variable	Abbreviation	Observation or reanalysis datasets	Time span	Horizontal resolution
Sea surface temperature	SST	OISSTv2	1982–2010	0.25° × 0.25°
		ERSSTv5	1901–2010	2° × 2°
		HadISSTv2	1901–2010	1° × 1°
Sea surface salinity	SSS	IAP_SSS	1950–2010	1° × 1°
		Argo_SSS	2005–2010	1° × 1°
Sea surface height	SSH	AVISO	1993–2010	0.25° × 0.25°
Ocean temperature	—	IAP_T	1950–2010	1° × 1°
Ocean salinity	—	IAP_S	1950–2010	1° × 1°
Atlantic Meridional Overturning Circulation	AMOC	RAPID array	200404–201012	—

The evolution of globally averaged SST over the past 110 years (1901–2010) is reproduced well in this century-long eddy-resolving simulation compared with three available observational datasets—namely, the Optimum Interpolation SST version 2 (OISSTv2^76,77^), the Extended Reconstructed SST version 5 (ERSSTv5^78^), and the Hadley Centre Sea Ice and Sea Surface Temperature dataset version 2 (HadISSTv2^79^), as shown in Fig. 1a. The simulated temporal evolution of globally averaged SST has high correlation coefficients with the three datasets: 0.96, 0.95 and 0.87 for OISSTv2, ERSSTv5 and HadISSTv2, respectively. The biases are negative, with a magnitude of around 0.1–0.5 °C. Additionally, the simulated result shows that the ocean surface temperature demonstrates interannual variabilities as observed, with a warming trend during 1901–2010. The simulated linear trends are comparable with those from the observations: 1.48 for OISSTv2 and 0.98 °C/century for this simulation during 1982–2010, and 0.74/0.34 for ERSSTv5/HadISSTv2 and 0.46 °C/century for this simulation during 1901–2010.

The surface salinity from Argo (Argo_SSS^80^) and IAP/CAS (IAP_SSS^81^) datasets are used to compare with our eddy-resolving simulation (Fig. 1b). The globally averaged ocean surface is fresher in the simulation, with relatively low temporal correlation coefficients: 0.70 and −0.20 for Argo_SSS and IAP_SSS, respectively. These low correlations may indicate large uncertainties for LICOM3’s surface freshwater fluxes, which have also been found in previous studies^40,46,47^.

The evolutions of averaged ocean temperature in the upper 2000 m during 1901–2010 from this century-long simulation and the IAP/CAS (IAP_T) datasets are shown in Fig. 1c. The temporal correlation coefficient between the two datasets is 0.95. In contrast to the negative bias of SST, the simulated ocean temperature averaged in the upper 2000 m shows a positive bias of around 1.5 °C, which is further illustrated in Fig. 3 in the next section. The simulated ocean temperature also has a small warming trend: 0.28 °C/century during 1901–2010 and 0.30 °C/century during 1950–2010, which is larger than that from IAP_T for the same period (0.19 °C/century).

Previous studies have pointed out that the warming upper ocean has led to a significant strengthening ocean circulation over the past two decades^22,23^. The ocean mean circulation in the upper 2000 m over the past several decades has accelerated, at a rate of 4.89 cm^2^/s^2^/century from 1960 to 1990 and 11.48 cm^2^/s^2^/century from 1990 to 2010 (Fig. 1d)^22^. Besides, significant variabilities on decadal and multidecadal time scales concurrently exist, showing relatively high values around 1940–1950 and 1995–2000 and low values around 1905–1915 and 1965–1975.

### Large-scale ocean circulation and variabilities

**Fig. 2:**
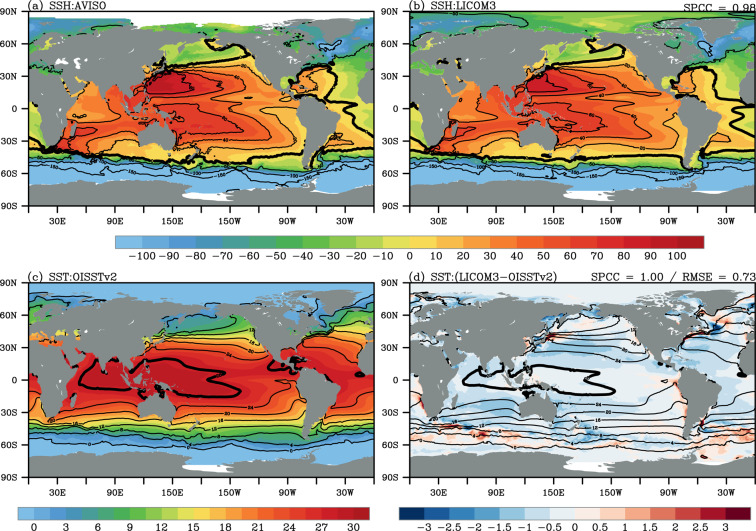
Mean sea surface height (SSH, cm) of (**a**) AVISO and (**b**) the LICOM3 simulation during 1993–2010. The spatial pattern correlation coefficient (SPCC) between the LICOM3 simulation and AVISO is noted in the top right of panel (**b**). The thick contours in (**a,b**) represent 0 cm. (**c**) Mean sea surface temperature (SST, °C) of OISSTv2 and the (**d**) biases of the LICOM3 simulation against OISSTv2 (shading) during 1982–2010. The contours in (d) are the mean SST in the LICOM3 simulation. The SPCC and root-mean-square error (RMSE) between the LICOM3 simulation and OISSTv2 are noted in the top right of panel (**d**). The thick contours in (**c,d**) represent 28 °C.

**Fig. 3:**
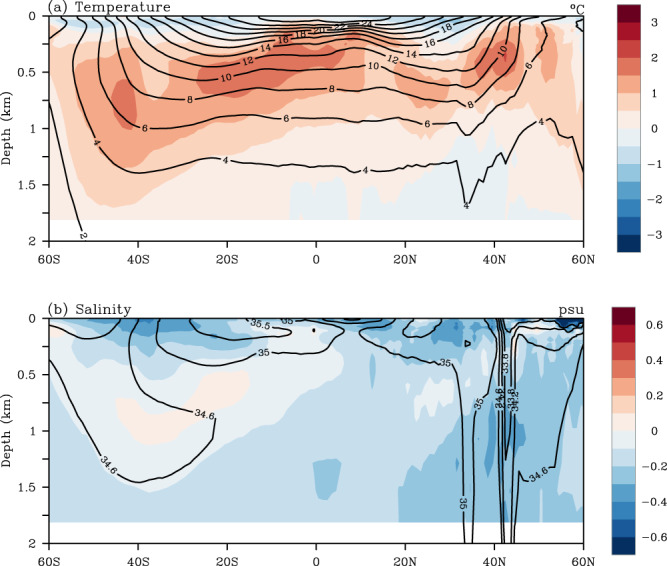
Mean zonally averaged global ocean (**a**) temperature (°C; contours) and (**b**) salinity (psu; contours) in the upper 2000 m ocean of the LICOM3 simulation and their biases (shading) against those from IAP/CAS datasets during 1950–2010.

**Fig. 4:**
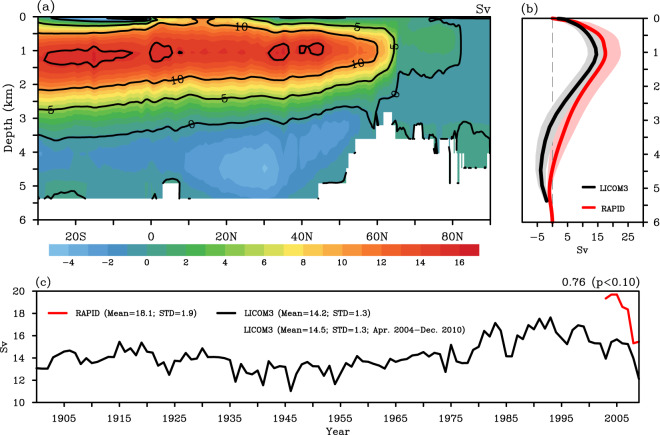
(**a**) Structure of the mean Atlantic Meridional Overturning Circulation (AMOC) stream function (Sv) calculated in the depth–latitude plane for the LICOM3 simulation from April 2004 to December 2010. (**b**) Mean depth profiles of the AMOC stream function (Sv) at 26.5°N for the LICOM3 simulation (black) and the RAPID array (red) with one standard deviation (STD; shading) from April 2004 to December 2010. (**c**) Yearly mean of the AMOC stream function (Sv) at 26.5°N for the LICOM3 simulation (black) during 1901–2010 and the RAPID array (red) from April 2004 to December 2010. The mean values and STDs are labeled in the legend. The temporal correlation coefficient and the corresponding *p*-value are noted in the top right of the panel.

**Fig. 5:**
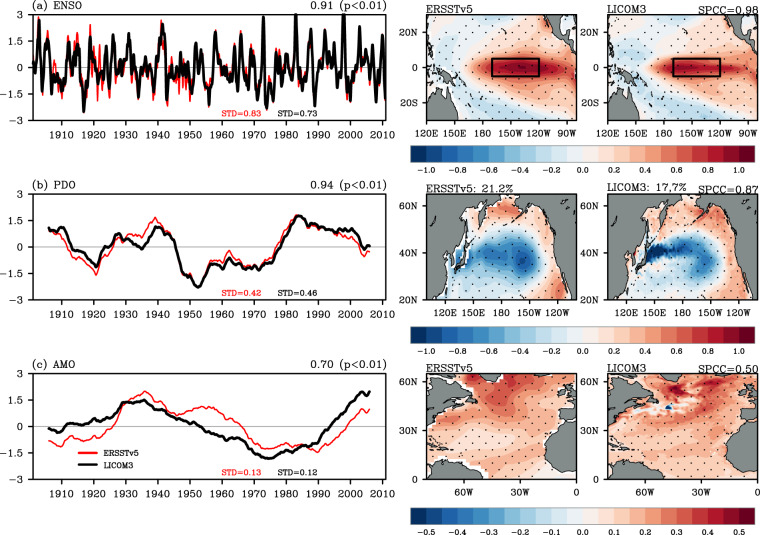
The time series (curves) and regressed spatial patterns for (**a**) El Niño–Southern Oscillation (ENSO; 3-month running average), (**b**) the Pacific Decadal Oscillation (PDO; 121-month running average), and (**c**) the Atlantic Multidecadal Oscillation (AMO; 121-month running average) for ERSSTv5 (red curves in the first and second column) and the LICOM3 simulation (black curves in the first and third column). The temporal correlation coefficients and the corresponding *p*-values are noted in the top right of each panel. The explained variances of the PDO are also noted in (**b**). The SPCCs between ERSSTv5 and the LICOM3 simulation are marked in the third column, calculated after the simulated regressed pattern has been interpolated into rectilinear grids, as in ERSSTv5.

This century-long simulation reproduces the large-scale ocean circulation well, such as the Atlantic Meridional Overturning Circulation (AMOC), both in terms of the climatological mean and the variability at interannual and decadal scales. The mean SSH and SST are evaluated against the satellite altimetry dataset and OISSTv2, respectively (Fig. 2). The mean zonally averaged vertical temperature and salinity are compared with those from the IAP/CAS dataset (Fig. 3). The simulated AMOC is validated against the RAPID array (RAPID/MOCHA/WBTS array^82,83^, Fig. 4). Finally, three leading climate modes on different temporal scales are compared with the results derived from ERSSTv5 (Fig. 5).

#### Climatology

SSH and SST are two valuable metrics of upper-ocean circulation^84^. Figure 2a,b give the mean SSH over the globe during 1993–2010 from the altimetry datasets and the simulation, respectively. The model realistically captures the main features of the basin-scale barotropic circulation, such as the positive SSH in the subtropical gyres in the two hemispheres and negative SSH in the region south of 55°S. The simulated and observed mean SSH demonstrate broad-scale agreement, with a spatial pattern correlation coefficient (SPCC) of 0.99. There are positive biases in the eastern tropical regions, subpolar North Pacific and North Atlantic, and Atlantic-Indian sectors in the Southern Ocean, and negative biases in the subtropical Pacific and the Indian Ocean (figure not shown). In most regions, the model biases of mean SSH are within 20 cm. Such biases are also seen in other eddy-resolving OGCMs^37–39,47^.

Figure 2c,d show the mean SST during 1982–2010 from OISSTv2 and the simulation. Overall, the observed large-scale pattern is well represented by the simulation, such as the Indo-Pacific warm pool (28 °C isotherm; the thick contour in Fig. 2c,d) and the equatorial cold tongue in the Pacific. A similar spatial pattern can be seen, with an SPCC of close to 1.0 between the simulation and OISSTv2. The global root-mean-square error (RMSE) of mean SST is 0.74 °C. Compared with OISSTv2, the simulated global mean SST shows a negative bias in most regions (shading in Fig. 2d). As in some eddy-resolving simulations^37–39^, the larger biases of mean SST mainly occur in the western boundary currents and their extensions and the Antarctic Circumpolar Current.

The simulation can also reproduce the realistic upper thermal and haline structures compared to the IAP/CAS datasets (Fig. 3) and other observational results from the World Ocean Atlas 2013^47,85^ and Argo profiles^85^. A significant warm bias in the thermocline dominates the positive bias of ocean temperature in the upper 2000 m (consistent with Fig. 1c). The cold bias can be found in the upper 100–200 m, which echoes the negative bias in Fig. 1a, and the lower 1500–2000 m in the Northern Hemisphere. Besides, the simulation reproduces a fresher upper ocean (Fig. 3b and Fig. 1b). The relatively larger model bias of zonally averaged salinity is located in the upper 100–200 m, south of 40°N. The model bias in the upper 2000 m is within 2 °C for the temperature and within 0.3 psu for the salinity.

#### Atlantic Meridional Overturning Circulation

The AMOC is one of the main inter-hemispheric upper overturning circulations^86–88^. The simulation realistically captures the vertical structure, with a positive upper cell extending to 3500 m and a negative lower cell (Fig. 4a). The upper cell exhibits maximum transport near 1000 m around 35°–40°N, and the lower cell has its maximum transport near 4500 m around 20°–25°N. Similar vertical structures of AMOC can be found in recent studies based on high-resolution models^47,87,88^. Furthermore, the variability of AMOC at 26.5°N remains consistent with observations from the RAPID array during 2004–2010 with a weaker strength (Fig. 4b,c). With a comparable depth of AMOC maximum value around 1 km, the simulation underestimates the depth of the return flow, demonstrating a relatively weaker and shallower upper cell and a stronger and deeper lower cell. This bias seems to be common in eddy-resolving OGCMs^38,47,87,88^.

The model also realistically reproduces AMOC’s temporal variability on the interannual timescale from April 2004 to December 2010, as compared with RAPID (Fig. 4c), with a temporal correlation coefficient of 0.73. During this period, the AMOC transports at 26.5°N for the simulation and RAPID are 14.5 ± 1.3 Sv and 18.1 ± 1.9 Sv, respectively, where the range represents one standard deviation (STD). The estimates of AMOC strength and variability also compare favorably with those from other eddy-resolving simulations^47,87,88^. Although with a weaker strength, this simulation can still record the notable decrease of around 3 Sv, about 20% of the AMOC strength over this period, reported in previous research^89,90^.

#### Modes of climate variability

Three major climate modes on different time scales [El Niño–Southern Oscillation^91,92^ (ENSO), Pacific Decadal Oscillation^93,94^ (PDO), and Atlantic Multidecadal Oscillation^95,96^ (AMO)] were used here to assess the performance in terms of large-scale basin modes (Fig. 5). The ERSSTv5^78^ dataset is used as the observational reference for the evaluation. In both the simulation and observation, the climatological monthly means were first removed to obtain the SST anomalies, and then the local linear trends were removed. Here, ENSO is described using the Niño3.4 index, which is defined as the area-weighted averages of SST anomalies over the Niño3.4 region (5°N–5°S, 170°–120°W)^91,92^. The PDO index is defined as the first principal component time series based on an empirical orthogonal function analysis of SST anomalies over the North Pacific (20°–70°N, 110°E–100°W)^93,94^. And for the AMO, its index is defined as the weighted average of SST anomalies over the North Atlantic after removing the global mean SST anomaly time series^95^.

The simulated Niño3.4 index demonstrates a significant interannual variability, as in ERSSTv5 (Fig. 5a). The major El Niño events, such as 1972/73, 1982/83 and 1997/98, are realistically reproduced^97^. The amplitude of ENSO in this simulation is comparable to that in ERSSTv5, with a significantly high temporal correlation coefficient (~0.91). The STDs for the three-month running averaged Niño3.4 index during 1901–2010 are 0.83 °C for the observation and 0.73 °C for the simulation. It is worth noting that the simulated and observed Niño3.4 index values match better after 1950 (temporal correlation coefficient of 0.95). In addition, the simulated STD of 0.82 °C during 1982–2010 is also very close to that (0.85 °C) from OISSTv2 (figure not shown). In the simulation and observation, the warming SST anomalies associated with El Niño events are located in the eastern tropical Pacific. A high SPCC (~0.98) between the simulation and observation is achieved. The smaller STD of Niño3.4’s evolution shown in this eddy-resolving simulation corresponds with the latest findings based on high-resolution OGCMs that the amplitude of ENSO event is suppressed in response to global warming^51,98^. The evaluation confirms that the resolving mesoscale oceanic processes in the tropical Pacific, such as the tropical instability waves, play a crucial role in regulating ENSO dynamics and its response to climate change^51,98,99^.

On the decadal scale, we chose the first leading mode in the North Pacific, i.e., the PDO, for validation (Fig. 5c,d). The evolution of the simulated PDO index matches that in ERSSTv5 very well, with a temporal correlation coefficient of 0.94. The simulated STD of PDO index is close to the observed one (0.46 and 0.42, respectively). Meanwhile, the simulated and observed PDO index values show synchronized variabilities, with a typical periodicity of 10–20 years^93,94^. This century-long simulation also replicates the anomalous cooling signals in the interior North Pacific, and warming along the Pacific coast (SPCC = 0.87) during the PDO positive phase. Meanwhile, a slightly smaller explained variance for the PDO is found in the simulation (17.7%) compared with ERSSTv5 (21.2%). In the simulation, because of its finer resolution than ERSSTv5, the relatively larger SST anomalies in the regressed pattern are located around the Kuroshio Extension with energetic mesoscale eddies. It is suggested that future investigations would benefit from this eddy-resolving simulation, which yields more consistent results that mesoscale variability can contribute significantly (by 10%–20%) to the large-scale variable climate in the North Pacific on the decadal scale in the Kuroshio Extension^100–102^.

The AMO index and the regressed pattern are shown in Fig. 5c to evaluate the multidecadal variability. The simulated and observed AMO are warm from the 1920s to the 1960s, cold from the 1970s to the early 1990s, and warm in the mid-1990s. The simulated AMO amplitude is close to the observed (0.12 and 0.13, respectively), and the temporal correlation coefficient is 0.70. Moreover, the coherent warm spatial pattern in the North Atlantic associated with the warm phase of the AMO shown in ESSTv5 is also reproduced, which is the same as reported in previously published research^95,96^. Unlike the simulation of ENSO and the PDO, the simulation has a smaller SPCC for the AMO, at 0.50. The differences in the regressed pattern between the simulation and ERSSTv5 appear around the jet axis of the Gulf Stream. We speculate that the extensive mesoscale variabilities in the Gulf Stream may have potential imprints on the SST anomalies’ response to the AMO, as shown by the eddy-like structures in the simulation. Recently, a growing number of studies have pointed out that eddy-resolving simulations are crucial to the simulation and projection of AMOC amplitude^87,88^. Since the AMO is tightly related to the AMOC amplitude, it is also highly likely that the finer resolution is essential for a better understanding of multidecadal dynamics in the North Atlantic. However, this speculative assertion is beyond the scope of the present study.

### Mesoscale variability

**Fig. 6:**
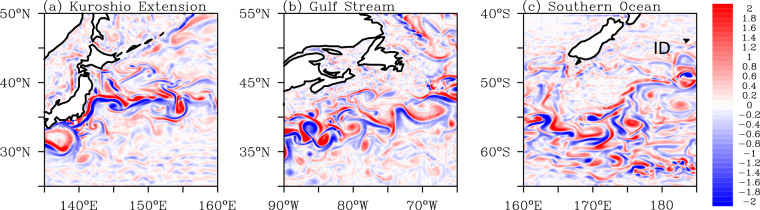
Daily snapshots of relative vorticity normalized by the Coriolis parameter over (**a**) the Kuroshio Extension (on 1 March 2000), (**b**) the Gulf Stream (on 1 March 2000), and (**c**) the Southern Ocean (on 1 October 2000).

**Fig. 7:**
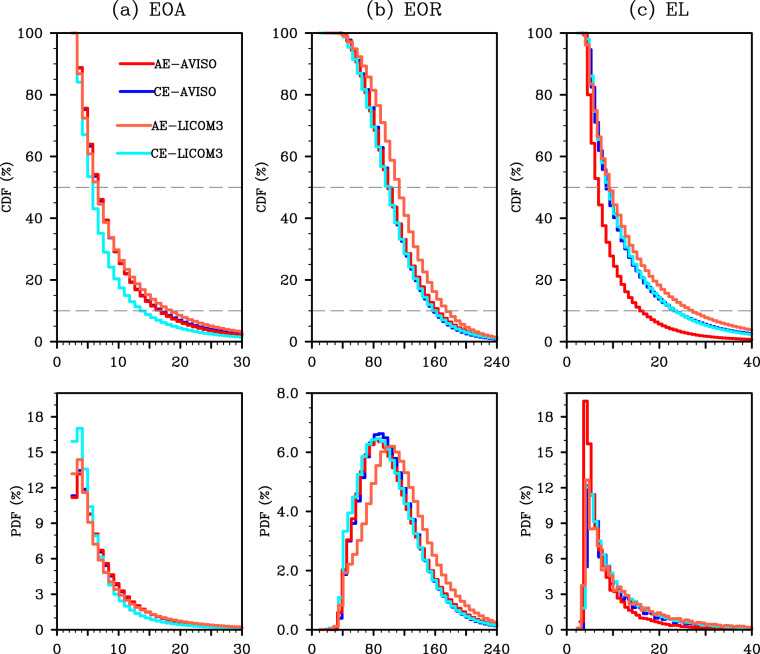
Upper-tail cumulative histograms (first row) and histograms (second row) for (**a**) the eddy occurrence amplitude (EOA, cm), (**b**) the eddy occurrence radius (EOR, km), and (**c**) the eddy lifetime (EL, weeks) during 1993–2010. The red and blue curves represent the results for observed anticyclonic and cyclonic eddies (CEs and AEs) based on AVISO, respectively. The orange and cyan curves represent CEs and AEs based on the LICOM3 simulation, respectively.

**Fig. 8:**
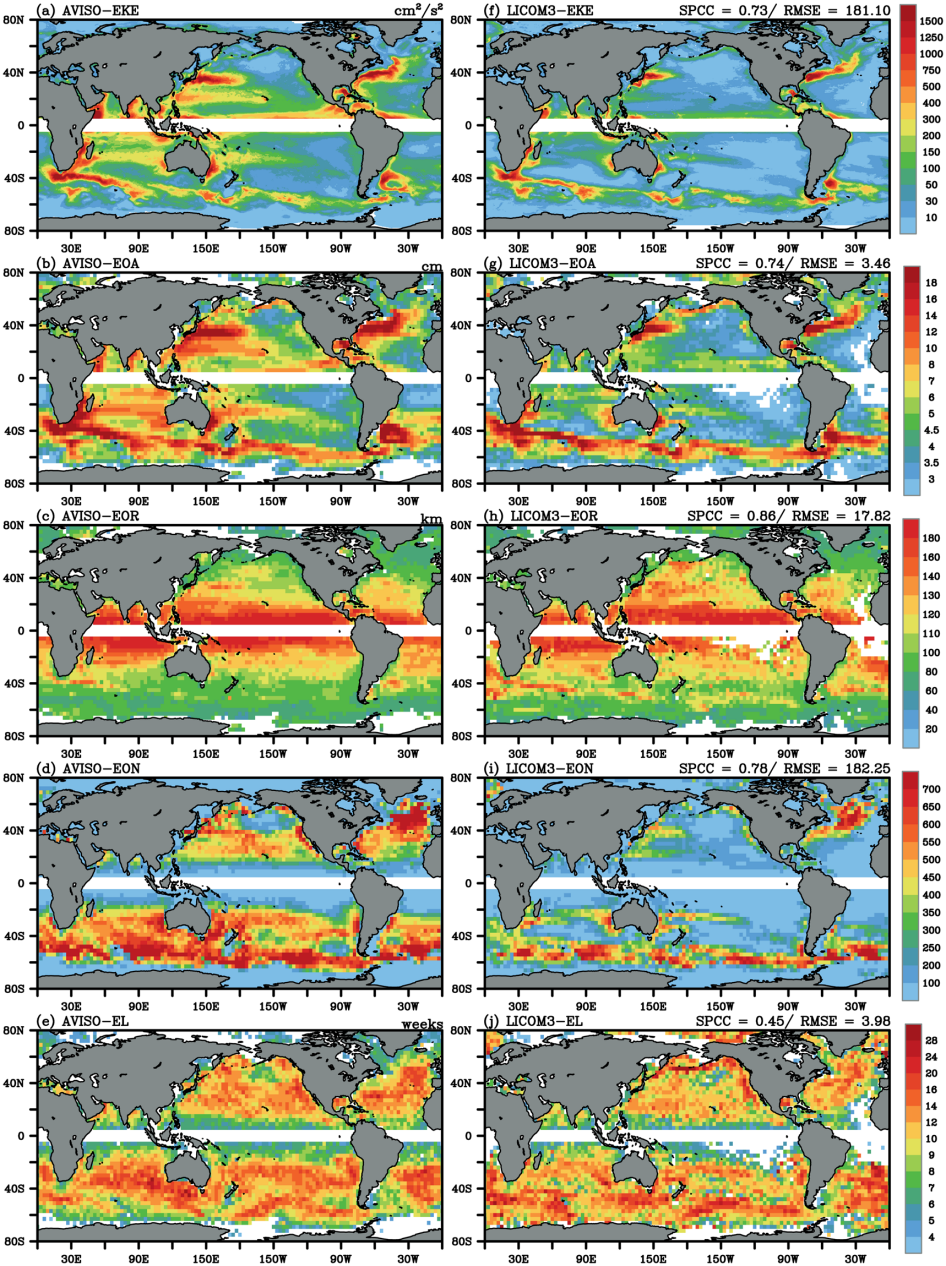
Mean of (**a**) the surface eddy kinetic energy (EKE, cm^2^/s^2^), (**b**) the eddy occurrence number (EON), (**c**) the EOA (cm), (**d**) the EOR (km), and (**e**) the EL (weeks) for AVISO during 1993–2010. (**f**–**j**) As in (**a**–**e**) but for the LICOM3 simulation. The SPCCs and RMSEs between the LICOM3 simulation and AVISO are noted in the top right of the panels.

Mesoscale eddies are ubiquitous in the ocean, with typical horizontal scales of less than 100 km and timescales on the order of months. Their random movement allows them to transport properties such as heat, salt, and carbon around the ocean^103,104^. With a horizontal resolution of at least 1/10°, these transient mesoscale eddies can be resolved in most of the open ocean^105^. Therefore, the performance of this eddy-resolving simulation in simulating global mesoscale eddies is evaluated with Eulerian metrics such as surface relative vorticity (Fig. 6) and eddy kinetic energy (EKE; Fig. 8), and Lagrangian metrics based on identified coherent mesoscale eddies (Figs. 7, 8 and Table 3).

**Table 3:** Global statistics of Lagrangian metrics based on identified coherent mesoscale eddies from AVISO (O, first column) and the LICOM3 simulation (M, second column) during 1993–2010, including the sum of the eddy generation number (EGN), eddy occurrence number (EON), the mean value (value in parentheses is the standard deviation) of eddy occurrence amplitude (EOA, cm), eddy occurrence radius (EOR, km) and eddy lifetime (EL, weeks) for cyclonic eddies (CEs) and anticyclonic eddies (AEs).

		AVISO	LICOM3 (1993–2010)	Ratio	LICOM3 (1901–2010)
**EGN**	**CE**	141,262	83,925	−40.6%	485,119
	**AE**	134,748	77,217	−42.7%	455,251
**EON**	**CE**	11,330,640	6,792,283	−40.1%	41,135,444
	**AE**	10,798,786	6,828,667	−36.8%	39,293,094
**EOA**	**CE**	8.2 (7.4)	6.8 (6.1)	−16.9%	7.5 (7.0)
	**AE**	8.1 (6.9)	8.4 (8.0)	4.5%	7.7 (7.2)
**EOR**	**CE**	101.4 (40.4)	99.1 (42.5)	−2.3%	103.0 (40.6)
	**AE**	103.6 (42.5)	110.9 (43.4)	7.0%	106.8 (42.6)
**EL**	**CE**	11.5 (10.5)	11.5 (10.9)	−0.2%	12.1 (11.1)
	**AE**	11.5 (10.0)	12.6 (11.5)	9.0%	12.3 (11.3)

The third column is the percentage model bias, calculated ${\rm{as}}\;\frac{M-O}{O}\times 100 \%$. The last column shows the results for the LICOM3 simulation during 1901–2010.

The active eddies over the two western boundary currents in the Northern Hemisphere and the Antarctic Circumpolar Current regions are clearly illustrated in the snapshots of the surface relative vorticity (Fig. 6). The relative vorticity fields indicate the strong large-scale currents and the vigorous mesoscale activities in these regions. This reflects the capability of this dataset to simulate mesoscale processes.

Therefore, the global coherent mesoscale eddies with lifetimes longer than 28 days are further identified and tracked. The simulated results of the Lagrangian eddy properties during 1993–2010 are quantified and compared with the satellite altimetry datasets. Table 3 gives the global statistics of eddy frequency for both cyclonic eddies (CEs) and anticyclonic eddies (AEs). In general, the simulated global mesoscale eddies agree reasonably well with the observed ones, but with less frequency. During the entire period of the satellite epoch, the eddy-resolving simulation generates 83,925 CEs and 77,217 AEs. The slight preference for CEs is well simulated, although there is a negative bias of around 40% against the observation (141,262 CEs and 134,748 AEs). The underestimation also happens for the eddy occurrence number (EON), but the bias for AEON (36.8%) is slightly smaller than that of CEON (40.1%). This underestimation of eddy frequency has been investigated and discussed in previous studies^106–108^.

In general, the intensity of global mesoscale eddies (shown by the eddy amplitude, radius, and lifetime) in this eddy-resolving simulation is also quantitatively similar to that in the satellite observations^71,72^ and other eddy-resolving simulations^106–108^. The global statistics in Table 3 show that the intensities of CEs (AEs) are underestimated (overestimated) by 0%–17% (4.5%–9%). The underestimation (overestimation) of CEs (AEs) is consistent with the results shown by the upper-tail cumulative histograms and histograms (Fig. 7). Taking the eddy occurrence amplitude (EOA; Fig. 7a) as an example, around 90% of the detected eddies have amplitudes less than 16 cm, and the eddy counts drop off rapidly with increasing amplitude. There is also a slight preference for AEs over CEs, with the eddy amplitude getting larger. As shown by the histograms, the frequency distribution of EOA shows a biased normal distribution. These findings hold for both the observation (red and blue curves in Fig. 7) and this eddy-resolving simulation (orange and cyan curves in Fig. 7). The evaluations of eddy occurrence radius (EOR) and eddy lifetime (EL) lead to a similar conclusion.

Additionally, the spatial patterns of simulated Eulerian and Lagrangian metrics are compared with those in the satellite dataset (Fig. 8). The spatial patterns and magnitudes of global mesoscale eddies are successfully simulated, with high SPCCs exceeding 0.70, except for EL, for which the SPCC is only 0.45. Similar to what we have learned from observation and previous research^71,72,106–108^, the high values of EKE and eddy amplitude simulated in this eddy-resolving simulation are located in the western boundary currents and their eastward extensions, and the Southern Ocean, suggesting extensive eddy activity in these regions of strong and unstable currents; the simulated spatial distribution of eddy radius shows an essentially monotonic decrease from about 180 km in the near-equatorial areas to about 60 km at 60° latitude; and for eddy frequency and EL, it is obvious that coherent eddies are simulated almost everywhere in the global ocean, with notable exceptions in the northeast and southeast Pacific Ocean, which we usually refer to as “eddy deserts”^71,72^. The spatial distributions for both CEs and AEs resemble Fig. 7 (figures not shown). The model–observation comparison suggests that the overall spatial distribution is similar to the observation but with a smaller intensity and lower eddy population. The relatively larger negative biases appear in the subtropical gyres.

## Data Availability

The forcing dataset for this century-long eddy-resolving simulation is available from https://www.ecmwf.int/en/forecasts/datasets/reanalysis-datasets/era-20c (last accessed: 29 May 2019). The source code of LICOM3 is available at https://github.com/yongqiangyu/FGOALS.git.
